# Imported diseases in travellers presenting to the emergency department after a stay in a malaria-endemic country: a retrospective observational study

**DOI:** 10.1186/s40794-023-00190-0

**Published:** 2023-02-20

**Authors:** Sofie Desmet, Liesbet Henckaerts, Sien Ombelet, Benjamin Damanet, Peter Vanbrabant

**Affiliations:** 1 Department of General Internal Medicine, KU Leuven, University Hospitals Leuven, Herestraat 49, Leuven, Belgium; 2 Department of Microbiology, Immunology and Transplantation, KU Leuven, University Hospitals Leuven, Herestraat 49, Leuven, Belgium; 3 Department of Laboratory Medicine, KU Leuven, University Hospitals Leuven, Herestraat 49, Leuven, Belgium; 4 School of Public Health, Free University of Brussels, Brussels, Belgium; 5 National Institute for Health and Disability Insurance (NIHDI), Brussels, Belgium

**Keywords:** Travel medicine, Communicable diseases, Imported, Malaria

## Abstract

**Background:**

We aimed to investigate the aetiology and outcomes of illnesses in patients presenting to an emergency department after travelling to a malaria-endemic country, in order to raise awareness of both tropical and cosmopolitan diseases.

**Methods:**

A retrospective chart review was performed for all patients who underwent blood smear testing for malaria at the Emergency Department of the University Hospitals Leuven from 2017 to 2020. Patient characteristics, results of laboratory and radiological examinations, diagnoses, disease course and outcome were collected and analysed.

**Results:**

A total of 253 patients were included in the study. The majority of ill travellers returned from Sub-Saharan Africa (68.4%) and Southeast Asia (19.4%). Their diagnoses fell into three major syndrome categories: systemic febrile illness (30.8%), inflammatory syndrome of unknown origin (23.3%) and acute diarrhoea (18.2%). Malaria (15.8%) was the most common specific diagnosis in patients with systemic febrile illness, followed by influenza (5.1%), rickettsiosis (3.2%), dengue (1.6%), enteric fever (0.8%), chikungunya (0.8%) and leptospirosis (0.8%). The presence of hyperbilirubinemia and thrombocytopenia increased the probability of malaria, with a likelihood ratio of 4.01 and 6.03, respectively. Seven patients (2.8%) were treated in the intensive care unit, and none died.

**Conclusion:**

Systemic febrile illness, inflammatory syndrome of unknown origin and acute diarrhoea were the three major syndromic categories in returning travellers presenting to our emergency department after a stay in a malaria-endemic country. Malaria was the most common specific diagnosis in patients with systemic febrile illness. None of the patients died.

**Supplementary Information:**

The online version contains supplementary material available at 10.1186/s40794-023-00190-0.

## Background

Until early 2020, international travel was increasing steadily. In 2018, more than 1.4 billion international trips were undertaken [1]. Illness during and after travelling to developing countries occurs frequently and results in 8% of all travellers seeking medical care [2]. The most frequent diagnoses are febrile illnesses, gastrointestinal disorders, dermatological disorders and respiratory tract infections [3, 4]. Malaria has been reported as the leading tropical cause of fever and the primary cause of death [5].

The emergency physician faces several challenges: most importantly, life-threatening diseases, such as malaria, must be recognised and treated adequately in a timely manner. Yet there is a knowledge gap in the diagnosis and management of patients with tropical diseases, leading to increased mortality and morbidity [6]. Moreover, the differential diagnoses of patients returning from the tropics are very broad, since not only tropical infections, but also cosmopolitan infectious and non-infectious diseases must be considered.

The aim of this study was to investigate the aetiology and outcomes of illnesses in patients presenting to the emergency department (ED) after a stay in a malaria-endemic country, in order to raise awareness of both tropical and cosmopolitan diseases.

## Methods

### Study design and patient population

This retrospective observational study was conducted at the University Hospitals Leuven, Belgium, a tertiary care referral hospital. A computer-assisted search for this chart review was performed to identify all patients who underwent diagnostic blood smear testing for malaria during a three-year period, from 28/02/2017 to 07/03/2020. We chose blood smear testing as a surrogate for illness after a stay in a malaria-endemic, as this test was part of the diagnostic workup for ill travellers returning from these countries [7, 8]. The files of all the selected patients were checked manually. Patients were excluded when they were younger than 16 years, when there was no evidence of a trip to a malaria-endemic country within the past year, when they were asymptomatic and when blood smears had been taken outside the ED in which was the study was conducted. Patients with repeated blood smears within the same illness episode were included in the analysis only once. The study protocol was approved by the Ethics Research Committee of University Hospitals Leuven (S64951).

### Medical records review

The following information was retrieved from the medical records: demographic data (age, gender, nationality and country of birth); travel history (travel destination, travel times and length of stay); reason for travel (tourism, work, visiting friends and relatives, volunteering, internship and recently arrived immigrant/expatriate); prophylaxis and adherence to malaria treatment (non-adherence was defined as not following the prescribed medication regimen); referral source (self-referred, general practitioner or another hospital or department within our hospital). Duration and onset of symptoms, intake of antimicrobial therapy before presentation, symptoms at presentation (fever (reported or measured in the ED), isolated fever (without focal signs or symptoms), respiratory symptoms, ear, nose and throat abnormalities, anorexia, diarrhoea, dysuria or urinary frequency, myalgia and arthralgia) were recorded. Relevant physical findings (e.g. lymphadenopathy, hepatosplenomegaly, skin lesions, abnormal lung auscultation,..) were collected from patient files. All available laboratory results (e.g. total blood cell count with differential, liver and kidney function test, blood smear, urinalysis, blood and stool cultures, serologic tests,…) were reviewed. Results of all performed radiological examinations were registered. Also records of follow-up care (during hospitalisation and until the final follow-up visit at the outpatient clinic) were reviewed for information on diagnosis, disease evolution and outcome.

Travel destinations were grouped in eight global regions corresponding to those defined for the GeoSentinel Surveillance Network: Sub-Saharan Africa, the Middle East and North Africa, Southeast Asia, South-Central Asia, Northeast Asia, Latin America and the Caribbean, North America and Australia, New Zealand and Oceania [9]. When different regions were visited, only the last visited destination was recorded.

### Outcome measures

Diagnoses were made in accordance with internationally recognised case definitions [10]. All diagnoses were confirmed by an infectious disease specialist (LH) and assigned to one of three categories: ‘confirmed diagnosis’ (demonstration of a pathogenic microorganism in a relevant specimen, or confirmed seroconversion to an infectious agent), ‘highly probable diagnosis’ (combination of clinical findings and a single serologic test result) or ‘clinical diagnosis’ (e.g. fever undisputedly due to a specific syndrome, such as erysipelas). Febrile episodes not meeting any precise etiologic or clinical diagnosis were classified as an ‘inflammatory syndrome of unknown origin’. In cases of more than one diagnosis, the principal diagnosis was retained. Hospitalisation (including length of stay) and mortality were recorded.

### Data analysis

Categorical variables are reported as totals and frequencies, and continuous variables are expressed as medians with interquartile ranges (IQR). We used 2 × 2 tables to calculate sensitivity, specificity, positive and negative likelihood ratios of signs, symptoms and laboratory findings. The confidence intervals (CI) for sensitivity, specificity and accuracy were 'exact' Clopper-Pearson confidence intervals.

The CI of the likelihood ratios were calculated using the log method [11]. Data were analysed using Medcalc Statistical Software version 20.011 (MedCalc Software, Ostend, Belgium).

## Results

### Patient characteristics

A total of 362 blood smears from the returning travellers in our ED were performed in the predefined period, and 109 blood smears were excluded from further analysis because of failure to meet the age criterion (*n* = 39), repeated blood smears from the same patient (*n* = 38), absence of travel to malaria-endemic country within the past year (*n* = 26), incomplete patient records (*n* = 4), blood smear taken outside the ED (*n* = 1) or being asymptomatic (*n* = 1).

The characteristics of the 253 patients who met the inclusion criteria are shown in Table 1. The median duration of travel was 18 days (IQR 10–28). Most patients presented at the clinic within the first month after their return (87%). The majority of ill travellers returned from Sub-Saharan Africa (68.4%) and Southeast Asia (19.4%), followed by Latin America and the Caribbean (6.3%), South-Central Asia (5.5%) and Northeast Asia (0.4%). Tourism (45.8%), work (22,1%) and visiting friends and relatives (20.9%) were the most frequent reasons for travel.

**Table 1:** Descriptive statistics (*N* = 253)

Age, y, median (IQR)	41 (30–53)
Sex, male, n (%)	143 (56.5)
Nationality, Belgian, total (%)	224 (88.5)
Duration of travel, days, median (IQR)^a^	18 (10–28)
Duration of symptoms before presentation ED, days, median (IQR)^b^	3 (1–5)
Time since end of travel to presentation at clinic, n (%)^c^	
< 1 week	129(51.0)
> 1–2 weeks	51(20.2)
> 2–3 weeks	25(9.9)
> 3–4 weeks	15(5.9)
> 1–2 mo	12 (4.7)
> 2–6 mo	13 (5.1)
> 6 mo	4 (1.6)
Geographic region visited, n (%)	
Sub-Saharan Africa	173 (68.4)
Southeast Asia	49 (19.4)
Latin America and Carribean	16 (6.3)
South-Central Asia	14 (5.5)
Northeast Asia	1 (0.4)
Travel motivation, n (%)	
Tourism	116 (45.8)
Work	56 (22.1)
Visiting friends and relatives	53 (20.9)
Volunteering	9 (3.6)
Internship	9 (3.6)
Recently arrived immigrant/expatriate	5 (2.0)
Other^d^	1 (0.4)
Unknown	4 (1.6)
Referral^e^	
General practitioner	108 (42.7)
Self-referred	95 (37.5)
Another hospital	10 (4.0)
Other^f^	3 (1.2)

*Abbreviations*: *IQR* Interquartile range, *ED* Emergency department, *mo* Month(s)

^a^ Missing data (*n* = 51), expatriates (*n* = 16) not included

^b^ Missing data (*n* = 17)

^c^ Missing data (*n* = 4)

^d^ Medical tourism in Belgium

^e^ Missing data (*n* = 37)

^f^ Other (other department in hospital, Institute for Tropical Medicine)

### Clinical findings

**Fig. 1:**
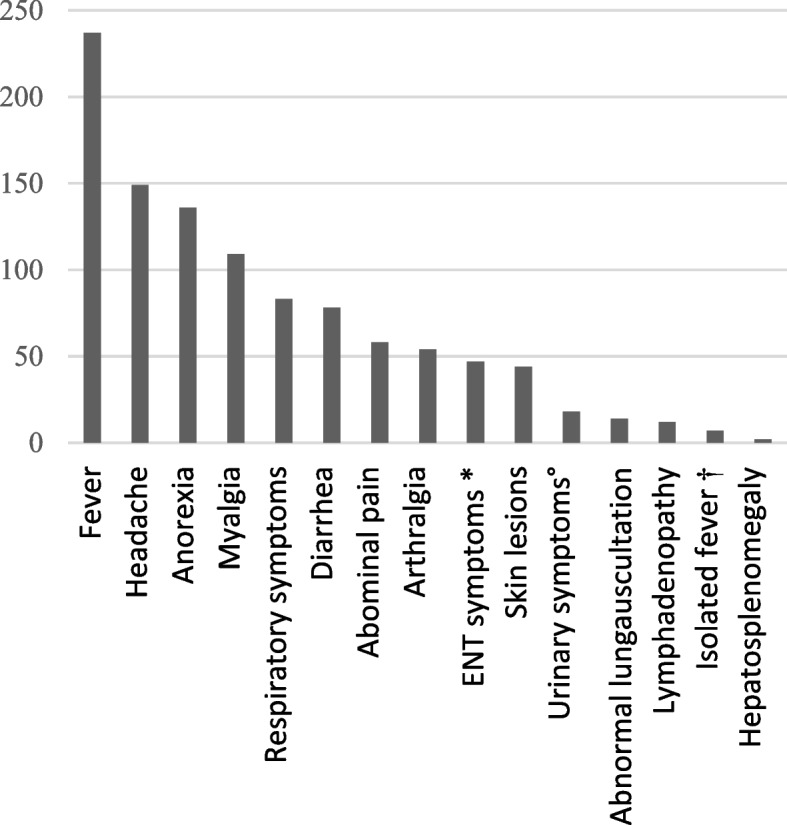
Symptoms and signs at presentation. * ENT symptoms: earache, sore throat, common cold, sinusitis. † Fever without focal signs or symptoms. ° Dysuria, urinary frequency

Frequencies of clinical signs and symptoms at presentation are shown in Fig. 1. Fever was present in 93.7% of the patients. Other frequently presenting symptoms included headache (58.9%), anorexia (53.8%), myalgia (43.1%), respiratory symptoms (32.8%) and diarrhoea (30.8%).

### Laboratory and radiology results

Blood cultures were obtained from 231 out of 253 patients and were positive in 5.2% (*n* = 12). Detected bacteria included *Salmonella* Paratyphi A (*n* = 1), *Salmonella* Paratyphi B (*n* = 1), *Salmonella* group B (*n* = 1), *Salmonella* group D (*n* = 1), *Escherichia coli* (*n* = 3), *Streptococcus agalactiae* (*n* = 1) and *Streptococcus pneumoniae* (*n* = 1). The other three positive cultures were contaminated with coagulase negative staphylococci (*n* = 1), *Acinetobacter Iwoffii* (*n* = 1) and *Corynebacterium* spp. (*n* = 1). Urine cultures turned positive in 5.2% (10/192). Stool cultures were positive in 13 out of 38 tests: isolated pathogens consisted of *Salmonella* spp. (6/13), *Shigella* spp. (5/13) and *Campylobacter* spp. (2/13). All 54 patients that were tested for HIV were negative. Only 12 (6.9%) of the 175 chest X-rays performed were abnormal, showing an infiltrate (*n* = 10), pleural effusion (*n* = 1) or pulmonary nodule (*n* = 1). Abdominal ultrasound was performed in 138 patients (54.5%) and showed abnormalities in 23.9% of the examinations, including splenomegaly (*n* = 10), hepatosplenomegaly (*n* = 6), hepatomegaly (*n* = 3), lymphadenopathy (*n* = 3), colitis (*n* = 7), signs of enteritis (*n* = 3), acute appendicitis (*n* = 1) and sedimentation at the bladder base (*n* = 1). In 1 patient, 2 abnormalities were detected (hepatosplenomegaly and lymphadenopathy).

### Diagnoses

Table 2 represents the syndromic groups and specified diagnoses by geographic region. Diagnoses were classified in 11 syndromic categories. Malaria and viral and bacterial infections were grouped under syndromic category of ‘systemic febrile illness’. The diagnoses of 72.3% of the 253 returning travellers fell into three major syndrome categories: systemic febrile illness (30.8%), acute diarrhoea (18.2%) and inflammatory syndrome of unknown origin (23.3%). The diagnosis of non-infectious origin was found in 9.9% of patients. Life-threatening diseases of non-infectious origin included pulmonary embolism (*n* = 2), subarachnoid haemorrhage (*n* = 1) and lymphoproliferative disorder (*n* = 1).

**Table 2:** Diagnosis by geographic region (*N* = 253)

	**Geographic region**					
**Diagnosis**	**Sub-Saharan Africa**	**Southeast Asia**	**South America and Caribbean**	**South Central Asia**	**Northeast Asia**	**All regions (%)**
**Systemic febrile illness**	61	12	2	3	0	78 (30.8)
Malaria						40 (15.8)
*P. falciparum*	30	0	0	0	0	30
*P. malariae*	2	0	0	0	0	2
*P. vivax*	1	1	0	1	0	3
*P. ovale*	4	0	0	0	0	4
Type unknown	1	0	0	0	0	1
Viral infection						23 (9.1)
Puumala virus (Hantavirus)	1	0	0	0	0	1
Dengue	0	2	1	1	0	4
CMV	0	0	1	0	0	1
EBV	1	0	0	0	0	1
Shingles	1	0	0	0	0	1
Influenza A	8	3	0	0	0	11
Influenza B	2	0	0	0	0	2
Chikungunya	1	1	0	0	0	2
Bacterial infection						15 (5.9)
Leptospirosis	0	2	0	0	0	2
Rickettsiosis^a^	7	1	0	0	0	8
Enteric fever	0	1	0	1	0	2
Tick borne relapsing fever	1	0	0	0	0	1
*E. coli* bacteraemia (unknown origin)	1	0	0	0	0	1
*S. agalactiae* bacteraemia	0	1	0	0	0	1
**Acute diarrhoea**						46 (18.2)
*Campylobacter* spp.	2	1	1	0	0	4
non-typhoidal *Salmonella* spp*.*	4	3	1	0	0	8
*Shigella* spp*.*	2	1	0	2	0	5
Acute diarrhoea of unknown origin	23	3	0	3	0	29
**Respiratory tract infection (no pathogen identified)**						13 (5.1)
Upper respiratory tract infection	2	0	0	0	0	2
Pneumonia	3	3	2	0	0	8
Bronchitis	1	1	0	0	0	2
Atypical pneumonia	1	0	0	0	0	1
**Abdominal infection**						1 (0.4)
Acute appendicitis	1	0	0	0	0	1
**ENT infection (no pathogen identified)**						12 (4.7)
Tonsillitis	5	0	1	1	0	7
Acute otitis media/externa	0	1	0	0	0	1
Herpangina	0	1	0	0	0	1
Acute sinusitis	1	0	0	0	0	1
Viral pharyngitis	1	0	0	0	0	1
Acute otitis media with mastoiditis	0	0	0	1	0	1
**Dermatological infection**						6 (2.4)
Bacterial skin/soft tissue infection	5	0	1	0	0	6
**Genitourinary infection**						10 (4.0)
Pyelonephritis	1	0	1	1	0	3
Cystitis	0	1	0	0	0	1
Acute prostatitis	1	1	0	0	0	2
Urosepsis	1	0	1	0	0	2
Pelvic abscess	1	0	0	0	0	1
Urinary tract infection	0	1	0	0	0	1
**Central nervous system infection**						2 (0.8)
Viral meningitis	2	0	0	0	0	2
**Parasitic infection**						1 (0.4)
Toxoplasmosis	1	0	0	0	0	1
**Inflammatory syndrome of unknown origin**	36	14	5	3	1	59 (23.3)
**Non-infectious diagnosis**	18	6	1	0	0	25 (9.9)

Systemic febrile illness = malaria, viral and bacterial infections

*CMV* Cytomegalovirus, *EBV* Epstein-Barr virus,* ENT* Ear nose throat

^a^ Typical eschar (7 of 8 patients) and/or positive IgG antibody titer to *Rickettsia conorii* (3 of 8 patients)

Malaria was detected in 40 patients (15.8%), all of them not taking malaria prophylaxis (*n* = 33) or non-adherent (*n* = 7) with the prophylaxis (early discontinuation). In our study 5/40 (12,5%) had a negative blood smear. In four of these patients the rapid antigen detection test (BinaxNOW™ Malaria test, Abbott, Chicago, USA) turned positive (presence of histidine-rich protein II detected). One patient needed a PCR test (performed in the reference lab at the Institute of Tropical Medicine (ITM)) for diagnosis. *Plasmodium falciparum* was found in 75% of the malaria cases. Four patients (4/30, 13%) with falciparum malaria met the WHO criteria for severe malaria (impaired consciousness *n* = 3, jaundice *n* = 1). The majority of patients diagnosed with malaria returned from Sub-Saharan Africa (95.0%) and their most frequently reported symptoms were fever (97.5%), headache (75.0%), myalgia (55.0%), anorexia (37.5%), arthralgia (25.0%), abdominal pain (20.0%) and diarrhoea (17.5%). In three patients with a malaria diagnosis (3/40, 7.5%), a co-infection was detected (1 rickettsiosis (positive IgG antibodies to *Rickettsia conorii)*, 1 acute hepatitis E and 1 influenza B).

The presence of hyperbilirubinemia and thrombocytopenia increased the likelihood of malaria, with likelihood ratios of 4.01 (95% CI, 2.17—7.38) and 6.03 (95% CI: 4.08 – 8.90), respectively (Table 3). Splenomegaly was observed in five of the 19 patients with malaria who underwent abdominal ultrasound.

**Table 3:** Summary of individual findings for Malaria

	Number of patients	Malaria number (%)	Sensitivity % (95% CI)	Specificity % (95% CI)	Positive LR (95% CI)	Negative LR (95% CI)
Symptoms						
Fever	253	40 (15.8)	97.50 (86.84—99.94)	7.04 (3.99—11.35)	1.05 (0.99—1.12)	0.36 (0.05—2.61)
Isolated fever ^a^	253	40 (15.8)	2.50 (0.06—13.16)	97.18 (93.97—98.96)	0.89 (0.11—7.17)	1 (0.95—1.06)
Headache	253	40 (15.8)	75.00 (58.80—87.31)	44.13 (37.35—51.08)	1.34 (1.08—1.66)	0.57 (0.32—0.99)
Resporatory tract symptoms	253	40 (15.8)	12.50 (4.19—26.80)	63.38 (56.52—69.86)	0.34 (0.15—0.79)	1.38 (1.18—1.61)
ENT symptoms	253	40 (15.8)	5.00 (0.61 -16.92)	78.87 (72.77—84.15)	0.24 (0.06—0.94)	1.20 (1.09—1.33)
Abdominal pain	253	40 (15.8)	20.00 (9.05—35.65)	76.53 (70.25—82.05)	0.85 (0.44—1.66)	1.05 (0.88—1.24)
Anorexia	253	40 (15.8)	37.50 (22.73—54.20)	43.19 (36.44—50.14)	0.66 (0.44—1.00)	1.45 (1.09—1.92)
Diarrhea	253	40 (15.8)	17.50 (7.34—32.78)	66.67 (59.90—72.96)	0.52 (0.26—1.06)	1.24 (1.04—1.47)
Dysuria/urinary frequency	253	40 (15.8)	7.50 (1.57—20.39)	92.96 (88.65—96.01)	1.06 (0.32—3.51)	1.00 (0.90—1.09)
Myalgia	253	40 (15.8)	55.00 (38.49—70.74)	59.15 (52.23—65.82)	1.35 (0.97—1.86)	0.76 (0.53—1.09)
Arthralgia	253	40 (15.8)	25.00 (12.69—41.20)	79.34 (73.28 -84.57)	1.21 (0.67—2.20)	0.95 (0.78—1.14)
Laboratory findings						
Anaemia^b^	253	40 (15.8)	17.50 (7.34—32.78)	84.51 (78.94—89.09)	1.13 (0.54—2.37)	0.98 (0.84—1.14)
Hyperbilirubinemia ^c^	246	40 (16.3)	35.00 (20.63—51.68)	91.26 (86.54—94.74)	4.01 (2.17—7.38)	0.71 (0.57—0.90)
Thrombocytopenia ^d^	250	40 (16.0)	77.50 (61.55—89.16)	87.14 (81.85—91.35)	6.03 (4.08—8.90)	0.26 (0.14—0.46)
Splenomegaly on ultrasound	138	19 (13.8)	26.32 (9.15—51.20)	90.76 (84.06—95.29)	2.85 (1.11—7.29)	0.81 (0.62—1.07)

*CI* Confidence interval, *LR* Likelihood ratio, *ENT* Ear nose throat

^a^ fever without focal signs or symptoms

^b^ Hemoglobin level below 12 g/dl in women and below 14 g/dl in men

^c^ Total bilirubin level exceeding 1.18 mg/dl

^d^ Platelet count below 150 × 10^^9^/l

### Outcomes

The majority of the 253 patients (55.7%) were treated in ambulatory care setting; 44.3% required admission to the hospital, and 2.8% were admitted to the intensive care unit. The median length of hospital stay was 4 days (IQR 3–6). None of the patients died.

## Discussion

This study focusses on characteristics and outcomes of ill returning travellers presenting to the emergency department after a stay in a malaria-endemic country.

Malaria (*n* = 40) was the most common specific diagnosis in the patients with systemic febrile illness, followed by influenza (*n* = 13) and rickettsiosis (*n* = 8). *Plasmodium falciparum*, the cause of severe (potentially lethal) malaria, was identified in 75.0% of the patients with malaria. Compared to the study of Siikamaki et al., who performed a similar retrospective study in the ED looking at febrile adults after travel to malaria-endemic areas, our data show a higher percentage of *Plasmodium falciparum* malaria (11.9% vs 3.5%) [8]. As the great majority of falciparum malaria occurs in the region Sub-Saharan Africa, this difference can be explained by the higher proportion of patients returning from this region in our study (68.4% vs only 41.8%). Our data are in concordance with data from Bottieau et al. (ITM), who included mainly ambulatory patients with fever after a stay in the tropics: sub-Saharan Africa was the most frequent region of exposure (68%) and falciparum malaria was recorded in 22.1% of patients [5]. Our study confirms that most cases of falciparum malaria in returning travelers occur when the prescribed regimen for chemoprophylaxis is not followed. Nevertheless, as no prophylactic regimen gives complete protection, malaria should always be excluded in patients with a relevant travel history up until months after their return, and both medical professionals and patients should be educated about this [12]. Although thrombocytopenia increases the likelihood for malaria, it should be used with caution when trying to determine if fever is due to malaria or to another acquired infection. Thrombocytopenia was also found in patients with viral (hantavirus, dengue, chikungunya, influenza A and varicella zoster virus) as well as bacterial (rickettsiosis and enteric fever) infections. Thirteen % of the patients in our study had severe falciparum malaria, compared to 7.7% of cases in a London registry and 25% in the Finnish ED study [8, 13].

Early diagnosis and treatment of malaria is of utmost importance, since delay in diagnosis is associated with fatal outcome [14]. When analyzing the history of the patients eventually having malaria, 5 patients mentioned previous clinical visits before malaria was considered in the differential diagnosis. For example, one patient presented after five days of illness (shivering and general weakness) at the general practitioner. No blood smear was performed and paracetamol was prescribed. On day nine of symptoms he presented with fever and change of consciousness at our emergency department with diagnoses of severe falciparum malaria. Admission at the intensive care was necessary.

It is important to stress that malaria may be acquired together with other pathogens that are endemic in the visited areas (e.g. bacteria, viruses, other parasites), and that symptoms can overlap. Therefore, additional diagnoses should always be considered, even when a diagnosis of malaria has already been made: a tropical co-infection may be present, may require specific therapy and may cause significant morbidity when left untreated [15]. In our study blood cultures were not taken in all patients, whereas this would have been more prudent. A recent retrospective study from Sweden on travellers diagnosed with malaria showed a low frequency of bacterial coinfection (positive blood cultures in 0.3% of patients (*Salmonella* Enteritidis and *E. coli*)) [16].

Although recognition and early treatment of life-threatening tropical diseases by the emergency physician is crucial, in our study only 22.5% of the patients had a ‘tropical’ diagnosis (additional file 1). This is higher to the study of Siikamäki et al. in which 10% of the patients suffered from a tropical infection, but lower than in the ITM study (39%) [5, 8]; the higher ITM percentage reflecting the selection of patients sent to ITM. The most frequent cosmopolitan infections we found were acute diarrhoea (18.2%), respiratory tract infections (5.1%) and ear, nose and throat infections (4.7%). Previous studies show that physicians should always remain vigilant to the possibility of a serious cosmopolitan bacterial infection (that can resemble tropical viral infection on clinical grounds) when facing a returning traveller, so that antimicrobial therapy can be started in time, awaiting results from additional testing [17].

When we looked at the prevalence of tropical disease according to geographical region of exposure, rickettsiosis (typical eschar (7 of 8 patients) and/or positive IgG antibody titer to *Rickettsia conorii* (3 of 8 patients) was almost exclusively seen after return from Sub-Saharan Africa (7 of 8 patients), especially South-Africa (6/8), based on epidemiological data presumably 6 cases of African tick-bite fever. Enteric fever was only found in Asia in our study (Southeast Asia [*n* = 1] and South-Central Asia [*n* = 1]). Dengue was diagnosed in 4 patients (1.6%), all after travels to Asia or Latin America. These data are in accordance with the GeoSentinel surveillance data, and emphasize the importance of the region of return when determining the prior probability for a specific disease [9].

For a large proportion of the travellers presenting at the ED (23.3%), the aetiology of illness remained unclear, which is consistent with other reports in and outside the ED (no specific diagnosis in respectively 25.1 and 24.4% of patients) [5, 8].

Mortality in returning travellers has been reported to range from 0.2 to 0.5% in other studies [4, 5, 7], and was mainly caused by falciparum malaria (case fatality ranging from 0.2 to 3% [14]), and occasionally by dengue, melioidosis, leptospirosis, enteric fever and *Strongyloides* hyperinfection syndromes [5, 18–21]. In our study none of the patients died.

### Study limitations

Since this is a single-centred study performed in a tertiary care hospital, there are restrictions on generalisability to other non-tertiary centres. We used the blood smear test as a surrogate for illness in the returning travellers after a stay in a malaria-endemic country. Theoretically we may have missed some patients that erroneously did not have a blood smear, but we believe this is unlikely since training on imported diseases is organised on a yearly basis in our centre and the threshold to request a blood smear is low. We were not able to collect reliable data on pre-travel vaccination status and so we did not include it on our study. Furthermore inpatients presenting with diarrhoea; parasitic testing was not routinely performed. The indication was evaluated case by case. In our study, there were no diagnoses of parasitic diarrhoea (e.g. giardiasis or amebiasis) and this could be an underestimation. Finally, this study discusses the patient population before the onset of the Covid-19 pandemic. For now, it remains unclear if the pandemic will have a permanent influence on international travel and cause a shift in the epidemiology of diseases in returning travellers.

## Conclusion

Our study confirms the broad differential diagnoses of illnesses in returning travellers presenting at the ED after a stay in a malaria-endemic country, and thus the need for continued vigilance in the ED physician. Both tropical and non-tropical, infectious and non-infectious causes should be taken into account. Whether they are tropical or non-tropical, potentially life-threatening diseases should be excluded or confirmed first. A thorough clinical examination, directed laboratory tests and knowledge about the prevalence of tropical disease according to the geographical region of exposure can help narrow down the possibilities.

## Supplementary Information

**Additional file 1.** Final diagnoses.

## Data Availability

The datasets used and/or analysed during the current study are available from the corresponding author on reasonable request.

## Abbreviations

ED: Emergency department
CI: Confidence intervals
ITM: Institute of Tropical Medicine Antwerp
