# Supplementary material for: Imported diseases in travellers presenting to the emergency department after a stay in a malaria-endemic country: a retrospective observational study
Source: Trop Dis Travel Med Vaccines. 2023 Feb 20;9:3. doi: 10.1186/s40794-023-00190-0 (PMC9939251; doi:10.1186/s40794-023-00190-0)
Supplement: Supplementary file 1 — Additional file 1. Final diagnoses. [file 40794_2023_190_MOESM1_ESM.docx]

| **Additional file 1**  **Final diagnoses.** | | |  |
| --- | --- | --- | --- |
| **Diagnosis** | | | **Number of patients (%)** |
| **Tropical diseases** | | | **57 (22.5)** |
| Malaria | | 40 |  |
| *P. falciparum* | 30 |  |  |
| *P. malariae* | 2 |  |  |
| *P. vivax* | 3 |  |  |
| *P. ovale* | 4 |  |  |
| type unknown | 1 |  |  |
| Dengue | | 4 |  |
| Chikungunya | | 2 |  |
| Rickettsiosis ° | | 8 |  |
| Enteric fever | | 2 |  |
| Tick borne relapsing fever | | 1 |  |
| **Cosmopolitan infections** | | | **112 (44.3)** |
| Acute diarrhoea | | 46 |  |
| *Campylobacter* spp. | 4 |  |  |
| non-typhoidal *Salmonella* spp. | 8 |  |  |
| *Shigella* spp. | 5 |  |  |
| Acute diarrhoea of unknown origin | 29 |  |  |
| Viral infections | |  |  |
| Puumala virus (Hantavirus) | 1 |  |  |
| CMV | 1 |  |  |
| EBV | 1 |  |  |
| Shingles | 1 |  |  |
| Influenza A | 11 |  |  |
| Influenza B | 2 |  |  |
| Leptospirosis | | 2 |  |
| *E. coli* bacteraemia (unknown origin) | | 1 |  |
| *S. agalactiae* bacteraemia | | 1 |  |
| Respiratory tract infection (no pathogen identified) | | 13 |  |
| Abdominal infection | | 1 |  |
| ENT infection (no pathogen identified) | | 12 |  |
| Bacterial skin/soft tissue infection | | 6 |  |
| Genitourinary infection | | 10 |  |
| Central nervous system infection | | 2 |  |
| Toxoplasmosis | | 1 |  |
| **Inflammatory syndrome of unknown origin** | | | **59 (23.3)** |
| **Non-infectious diagnosis** | | | **25 (9.9)** |

CMV cytomegalovirus, EBV Epstein-Barr virus, ENT, ear nose throat

° Typical eschar (7 of 8 patients) and/or positive IgG antibody titer to *Rickettsia conorii* (3 of 8 patients)
